# Supplementary material for: Rapid cloning-free mutagenesis of new SARS-CoV-2 variants using a novel reverse genetics platform
Source: bioRxiv. 2023 Sep 11:2023.05.11.540343. Preprint. [Version 4] doi: 10.1101/2023.05.11.540343 (PMC10245781; doi:10.1101/2023.05.11.540343)
Supplement: Supplement 1 [file media-1.pdf]

Supplementary Materials for:

Rapid cloning-free mutagenesis of new SARS-CoV-2 variants using a novel reverse genetics platform

Enja Kipfer, David Hauser, Martin J. Lett, Fabian Otte, Lorena Urda, Yuepeng Zhang, Christopher M. R. Lang, Mohamed Chami, Christian Mittelholzer, Thomas Klimkait

Figure S1. Clonal virus populations and reconstitution efficiency

A Schematic representation of the workflow to generate clonal virus populations. Right after transfection, cells were diluted to less than 0.5 virus-producing cells/well in 96-well plates. 7 days post-transfection, the supernatant was transferred onto Vero E6-TMPRSS2 cells. Plates were observed until a CPE became apparent.

B Clonal virus populations arising from a single virus-producing cell were identified after supernatant transfer onto Vero E6-TMPRSS2 cells. CPE of infectious virus was assessed by microscopy and virus was collected for further analysis. Thereafter, plates were fixed and stained for the fast enumeration of positive wells (in light blue).

Figure S2. CLEVER primer design for direct mutagenesis

Shown are the different approaches of primer design in order to guarantee a 100 bp overlap between the neighboring fragments. Primers can either be separated in distance to ensure homology regions or additional nucleotides must be added to the 5' end of the primer. Small nucleotide changes, deletions or insertions can directly be introduced by adjusting the primer annealing sites and the homology regions, as depicted.

Figure S3. Cloning-free rescue of chimeric virus

A In addition to the chimeric viruses described in Figure 3, the genetic background (region outside of S) was replaced by the sequence of Omicron BA.1 or Omicron BA.5 and combined with a heterologous S sequence of Wuhan, Omicron BA.1 or BA.5, respectively.

B Infectious chimeric virus was rescued and assessed via CPE formation on Vero E6 cells. Scale bar represents 100 µm.

C The fragments were directly amplified by one-step RT-PCR from viral RNA of Wuhan, Omicron BA.1 and Omicron BA.5. Eight fragments were amplified to prove high flexibility in exchanging fragments.

D Scheme for the rapid distinguishment between Wuhan, Omicron BA.1, BA.5 and XBB.1.5 variants. Indicated regions (S or M) were Sanger sequenced to discriminate variants or confirm chimeric viruses. Amino acids are highlighted in yellow for the clear identification of the S gene variant and/or the background (within M).

Figure S4. Cloning-free rescue of CHIKV and DENV

A Schematic representation of the CHIKV genome and the design for the cloning-free rescue. The genome was divided into three overlapping fragments and a silent SNP was introduced by PCR (red asterisk).

B and C Successful recombination of the four PCR products (**B**) within the eukaryotic cell leads to a circular product and virus production (**C**, CPE on BHK-21 cells for rCHIKV and negative control).

D Schematic representation of the DENV genome and the design for the cloning-free rescue. The genome has been divided into two overlapping fragments and a silent SNP has been

introduced by PCR (red asterisk). The protocol has been tested on two different clinical isolates (DENV1 and DENV3), whereas two different recombination sites were tested for DENV1 (rDENV1-A and rDENV1-B, only one schematically represented).

E and F Overlapping PCR products (**E**) were transfected into BHK-21 cells and CPE (**F**) was assessed on VeroE6-TMPRSS2 cells (rDENV1-A, rDENV1-B, rDENV3 and negative control).

Scalebar is 100 μm in **C** and **F**.

Table S1. Infectivity assessment of recombinant virus on Vero E6 cells.

Vero E6 cells were infected with the indicated recombinant virus and pictures were taken 3 days post infection. Of note, the virus was not titrated and the development of CPE is not quantitative. Scale bar represents 100 µm.

Table S2. Genomic characterization of recombinant SARS-CoV-2 virus based on NGS data. Mutations with a relative abundance of > 10 % in the entire virus population are listed.

Table S3. Homology regions successfully used for recombinant SARS-CoV-2, CHIKV and DENV1/DENV3.

Length, GC content, and the hypothetical annealing temperature (according to OligoCalc, salt-adjusted) are listed. Note that homology regions were chosen independently from GC content or annealing temperature and values are only listed for completion.

Table S4. Sanger sequencing data of the region of interest for the generated mutant rSARS-CoV-2, rCHIKV and rDENV1/rDENV3.

Table S5. Primer tables.

Table S6. PCR settings for individual fragments.

Supplementary figure 1: Clonal virus populations and reconstitution efficiency

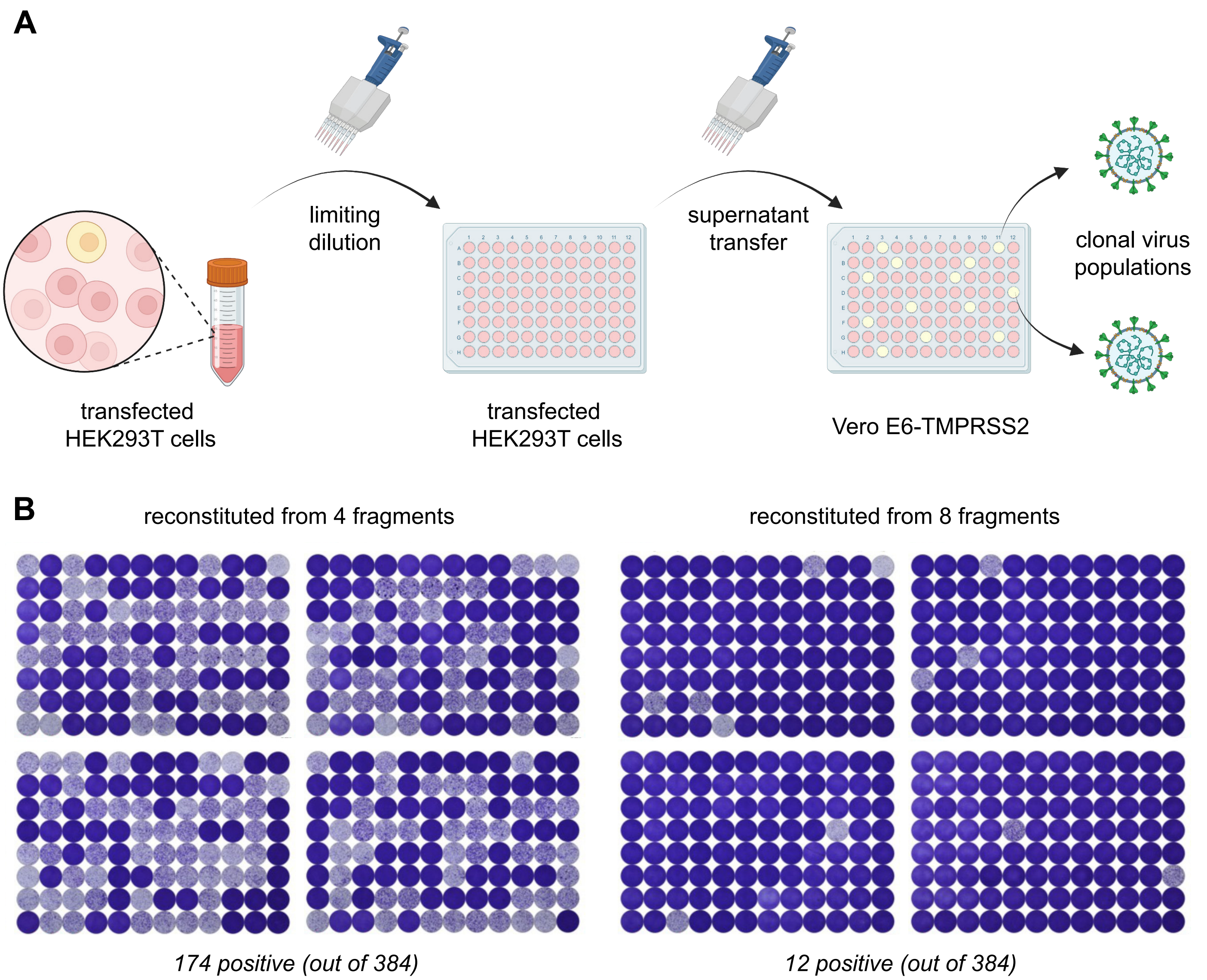

Supplementary figure 2: CLEVER primer design for direct mutagenesis

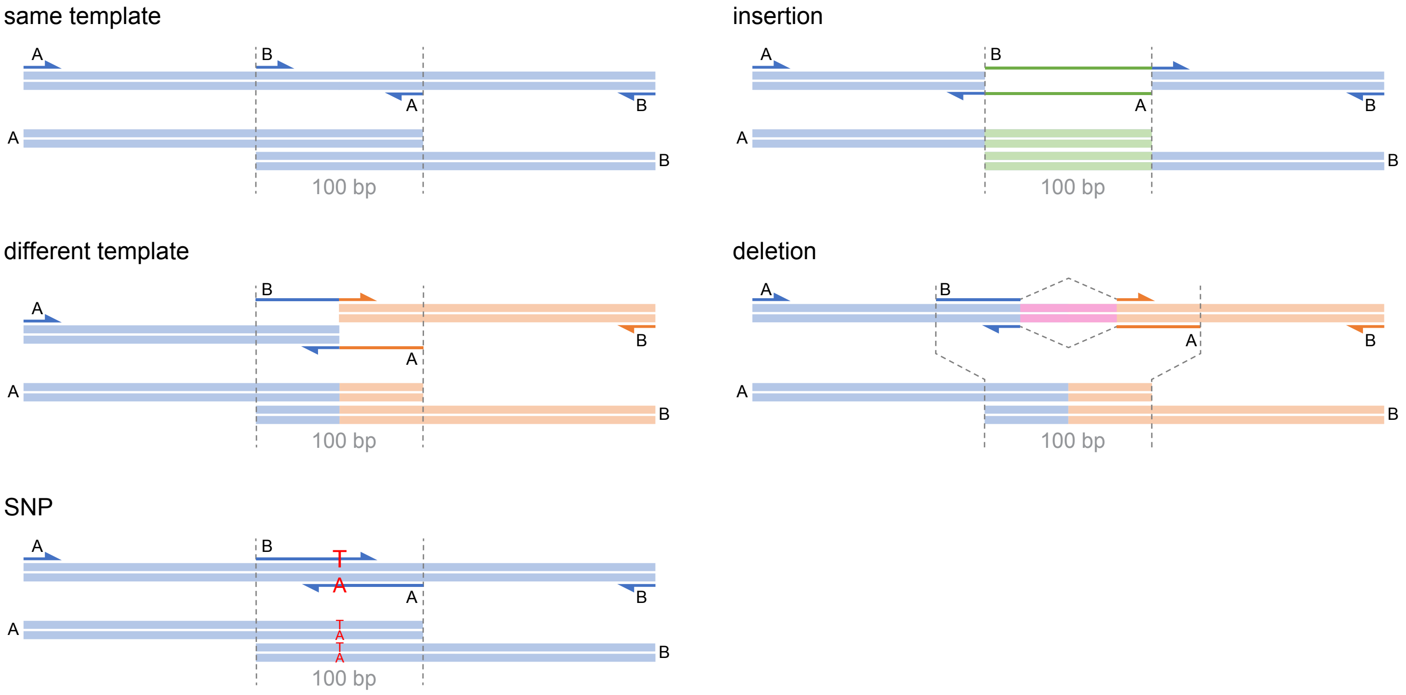

Supplementary figure 3: Cloning-free rescue of chimeric virus

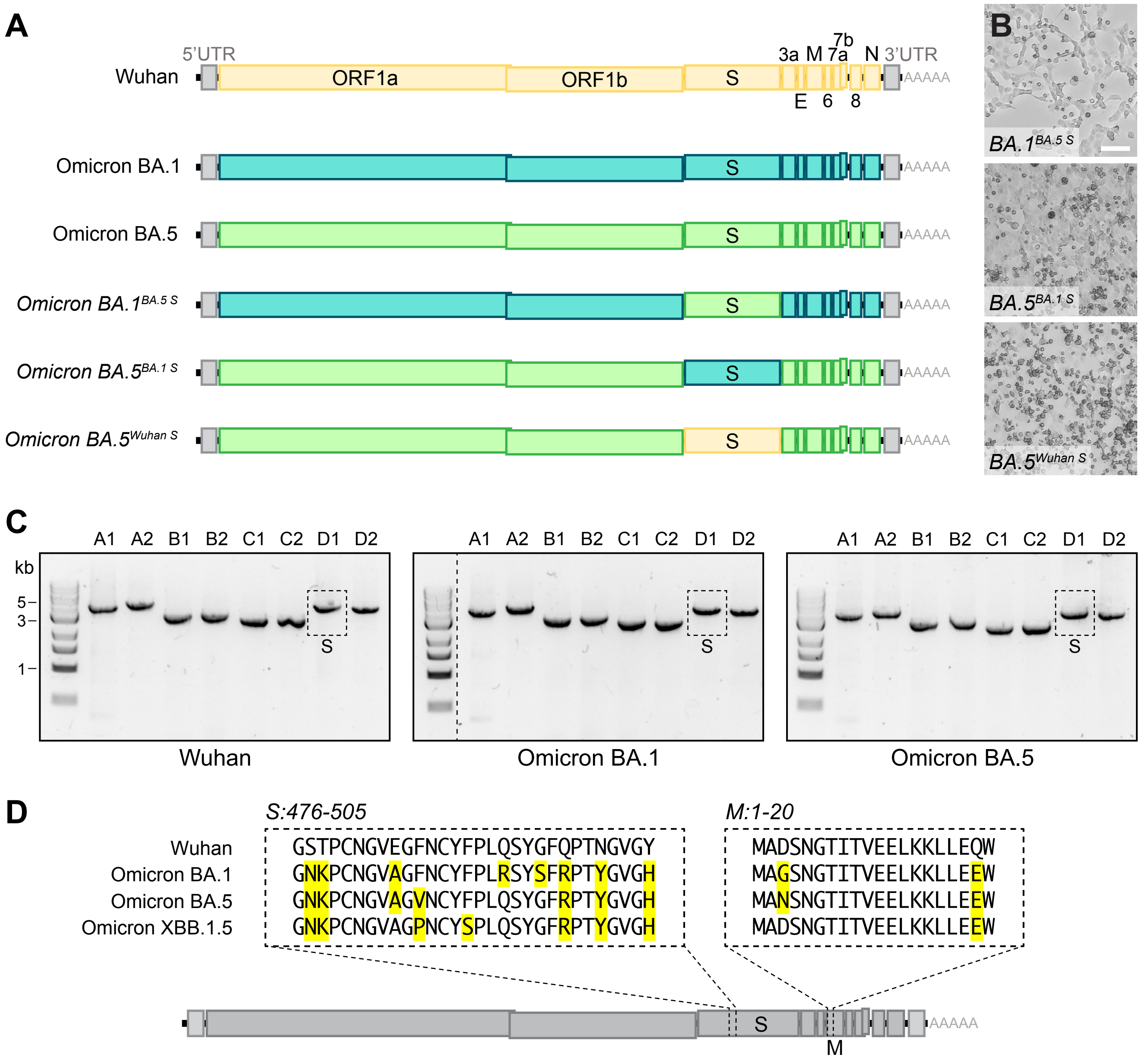

Supplementary figure 4: Cloning-free rescue of CHIKV and DENV

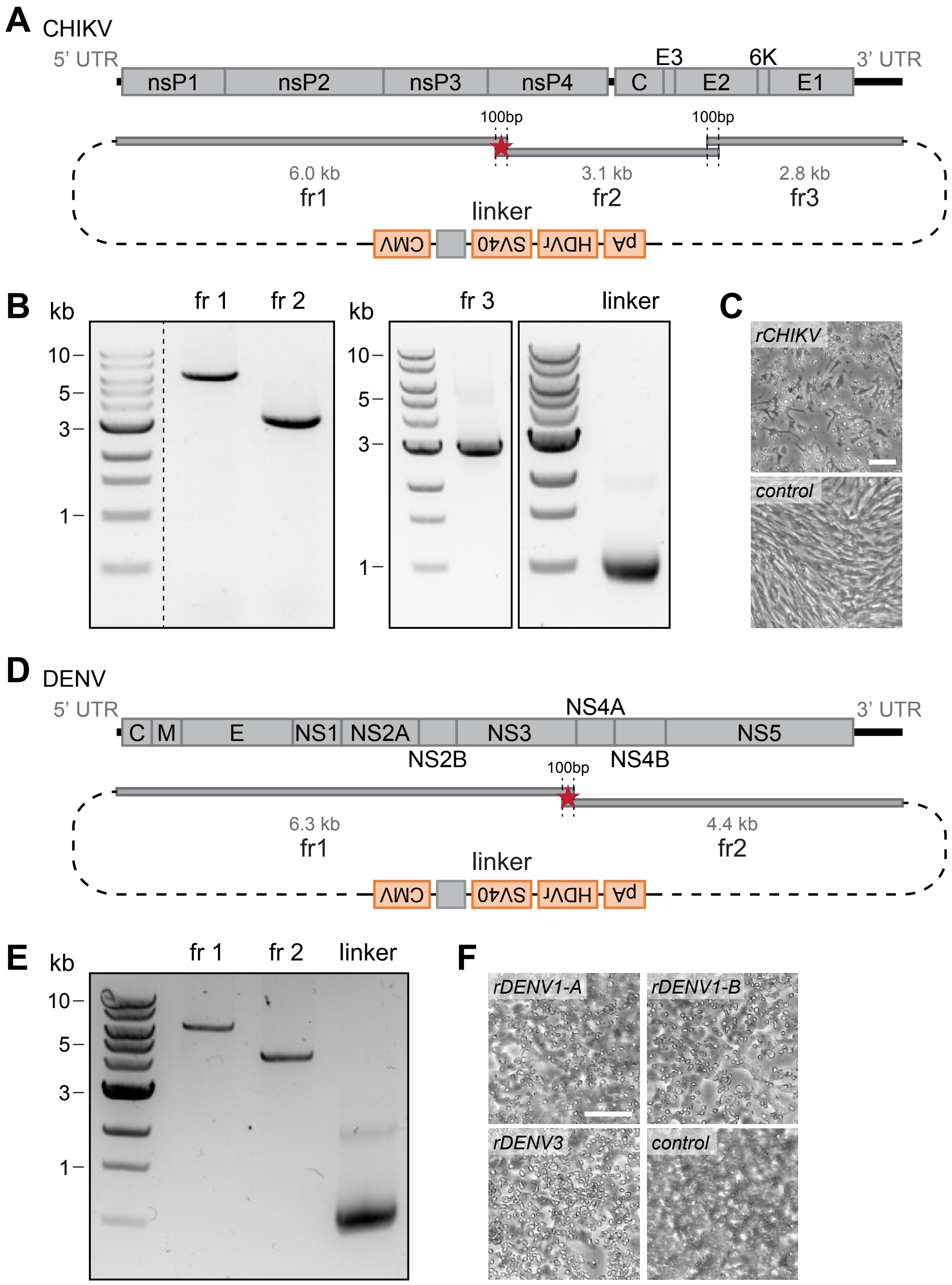

Table S1. Infectivity assessment of recombinant virus on Vero E6 cells.

Number of fragments	Transfection method	Name	Cell line transfected	3 days post infection on Vero E6	Description
-	-	Vero E6 negative control	-	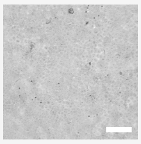	Uninfected Vero E6 cells.
8	electroporation	rCOV2-8fr	HEK293T	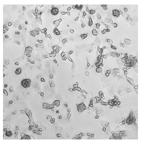	Rescue from eight fragments.
4	electroporation	rCOV2-4fr	HEK293T	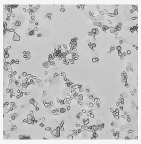	Rescue from four fragments.
4	electroporation	rCOV2-293	HEK293	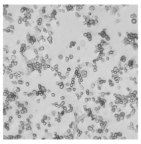	Rescue from HEK293.
4	electroporation	rCOV2-CHO	CHO	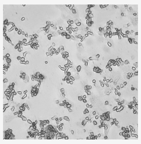	Rescue from CHO.
4	electroporation	rCOV2-BHK	BHK-21	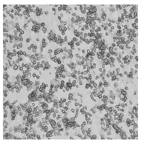	Rescue from BHK-21.
4	electroporation	rCOV2-A549	A549-ACE2	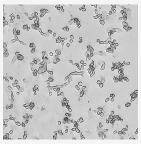	Rescue from A549-ACE2.
4	jetPRIME	rCOV2-jP	HEK293T	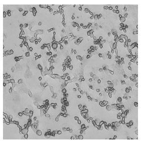	Rescue after transfection with jetPRIME.
4	Lipofectamine-3000	rCOV2-3000	HEK293T	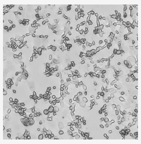	Rescue after transfection with Lipofectamine-3000.
4	Lipofectamine-LTX	rCOV2-LTX	HEK293T	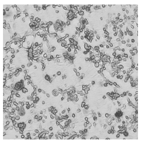	Rescue after transfection with Lipofectamine-LTX.

5	electroporation	rCOV2-500bp	HEK293T	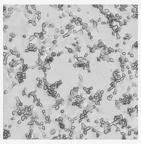	Rescue including one fragment to be only 500 bp in length.
5	electroporation	Wuhan$^{BA.1\ S}$	HEK293T	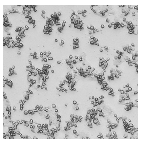	Chimeric virus, Omicron BA.1 S gene in the background of Wuhan.
5	electroporation	Wuhan$^{BA.5\ S}$	HEK293T	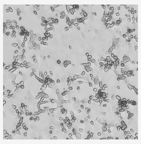	Chimeric virus, Omicron BA.5 S gene in the background of Wuhan.
5	electroporation	rCOV2-N501Y	HEK293T	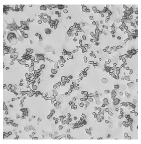	Direct mutagenesis, incorporation of N501Y substitution in the S gene.
5	electroporation	rCOV2-G614D	HEK293T	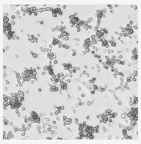	Direct mutagenesis, incorporation of G614D substitution in the S gene.
5	electroporation	rCOV2-∆ORF3a	HEK293T	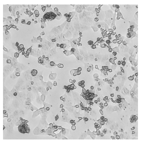	Direct mutagenesis, deletion of ORF3a.
5	electroporation	rCOV2-ORF8-FLAG	HEK293T	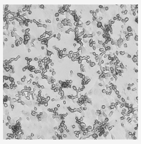	Direct mutagenesis, incorporation of a 3x-FLAG tag at the C-terminal of ORF8.
9	electroporation	rCOV2-link-Wuhan	HEK293T	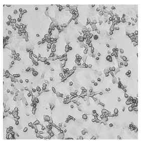	Rescue from RNA directly using the linker fragment.
9	electroporation	rCoV2-link-Omicron BA.5$^{BA.1\ S}$	HEK293T	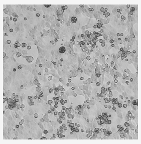	Rescue of chimeric virus from RNA directly using the linker fragment. Omicron BA.1 S gene in the background of Omicron BA.5.
9	electroporation	rCoV2-link-Omicron BA.1$^{BA.5\ S}$	HEK293T	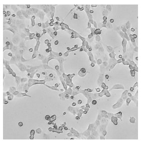	Rescue of chimeric virus from RNA directly using the linker fragment. Omicron BA.5 S gene in the background of Omicron BA.1.
9	electroporation	rCoV2-link-Omicron BA.5$^{Wuhan\ S}$	HEK293T	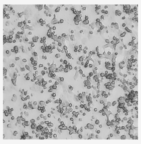	Rescue of chimeric virus from RNA directly using the linker fragment. Wuhan S gene in the background of Omicron BA.5.

Table S2. Genomic characterization of recombinant SARS-CoV-2 virus based on NGS data. Mutations with a relative abundance of > 10 % in the entire virus population are listed.

ID	position							"N" reads*	origin of template
	13551	21003*	21621*	21638*	25768*	28540*	28967*		
1		K	M	Y	M				T15324C
2			M	Y	M			5979, 9628, 9629, 20231, 20525, 20526	T15324Y
3			M	Y	M	M	M		T15324Y
4			M	Y	M	M			T15324Y
5			M	Y	M	M		5979, 5980, 9628, 9629, 20525, 21002, 21003	
6		K	M	Y	M	M	M	5979, 5980, 9628, 9629, 20525, 20526	T15324Y
7	C to T (13 %)		M	Y	M	M		9628, 9629	T15324Y
8		K	M	Y	M	M		5979, 9628, 9629, 20231, 20232, 20526	T15324Y
	orf1ab		*S*		*orf3a*	*N*			

* Ambiguities or "N" stretches occuring as clusters were not considered as a randomly introduced mutation during the PCR-amplification step but rather as technical artefact or culture adaptation of the virus.

ID	position							
	29**	***30*****	***79***	***259***	***263***	***264***	**550**	**12464**
clonal 1								
clonal 2	*A to G*	*A to G*	*A to G*	*A to G*	*A to G*	*A to G*	A to G	
clonal 3								
clonal 4								
clonal 5								G to T
	5'UTR						*orf1ab*	

*** low coverage (20 reads)*

ID	position														
	3561	5834	6067	18750	18897	19097	20429	20763	20855	20881	21137	21777	25101	27787	28720
RNA clonal 1		T to C		T to C							A to G		A to G	T del	C to T
RNA clonal 2							C to T	A to G	G to C	G to A					
RNA clonal 3	T to C		T to G		T to C	C to T						G to A			
	orf1ab											*S*		*orf7b*	*N*

Table S3. Homology regions successfully used for recombinant SARS-CoV-2, CHIKV and DENV1/DENV3. Length, GC content as well as the hypothetical annealing temperature (according to OligoCalc, salt-adjusted) are listed. Note that homology regions were chosen independently from GC content or annealing temperature and values are only listed for completion.

	Length (bp)	G/C (%)	Annealing (°C)	Sequence
SARS-CoV-2	100	30	83	GCACTTAAGGGTGGTAAAATTGTTAATAATTGGTTGAAGCAGTTAATTAAAGTTACACTTGTGTTCCTTTTTGTTGCTGCTATTTTCTATTTAATAACAC
	100	38	86.3	TATAACTCAAATGAATCTTAAGTATGCCATTAGTGCAAAGAATAGAGCTCGCACCGTAGCTGGTGTCTCTATCTGTAGTACTATGACCAATAGACAGTTT
	100	43	88.3	TACAGCTGTTTTAAGACAGTGGTTGCCTACGGGTACGCTGCTTGTCGACTCAGATCTTAATGACTTTGTCTCTGATGCAGATTCAACTTTGATTGGTGAT
	102	35	85.2	TGTGTTGAAGAAGTTACAACAACTCTGGAAGAAACTAAGTTCCTCACAGAAAACTTGTTACTTTATATTGACATTAATGGCAATCTTCATCCAGATTCTGCC
	93	43	87.7	CACAGGGACTACTCCCACCCAAGAATAGCATAGATGCCTTCAAACTCAACATTAAATTGTTGGGTGTTGGTGGCAAACCTTGTATCAAAGTAG
	107	36	86	TTTACAAGTCTTGAAATTCCACGTAGGAATGTGGCAACTTTACAAGCTGAAAATGTAACAGGACTCTTTAAAGATTGTAGTAAGGTAATCACTGGGTTACATCCTAC
	100	45	89.2	CAGTCAGCACCTCATGGTGTAGTCTTCTTGCATGTGACTTATGTCCCTGCACAAGAAAAGAACTTCACAACTGCTCCTGCCATTTGTCATGATGGAAAAG
	100	46	89.6	GGAACTGTAACTTTGAAGCAAGGTGAAATCAAGGATGCTACTCCTTCAGATTTTGTTCGCGCTACTGCAACGATACCGATACAAGCCTCACTCCCTTTCG
	100	34	84.6	CCACGCGAACAAATAGATGGTTATGTCATGCATGCAAATTACATATTTTGGAGGAATACAAATCCAATTCAGTTGTCTTCCTATTCTTTATTTGACATGA
	102	37	86	TGCTAGGGAGAGCTGCCTATATGGAAGAGCCCTAATGTGTAAAATTAATTTTAGTAGTGCTATCCCCATGTGATTTTAATAGCTTCTTAGGAGAATGACAAA
	119	40	88.4	GTTTAGTGAACCGTATATTAGGTTTATACCTTCCCAGGTAACAAACCAACCAACTTTCGATCTCTTGTAGATCTGTTCTCTAAACGAACTTTAAAATCTGTGTGGCTGTCACTCGGCTG
	100	34	84.6	AAGGTTTTAATTGTTACTTTCCTTTACAATCATATGGTTTCCAACCCACTAATGGTGTTGGTTACCAACCATACAGAGTAGTAGTACTTTCTTTTGAACT
	80	43	86.3	AACACCAGGAACAAATACTTCTAACCAGGTTGCTGTTCTTTATCAGGGTGTTAACTGCACAGAAGTCCCTGTTGCTATTC
	100	43	88.3	ACGACTCTGAGCCAGTGCTCAAAGGAGTCAAATTACATTACACATAAGCACAAGCTGATGAGTACGAACTTATGTACTCATTCGTTTCGGAAGAGACAGG
	84	43	86.8	GGCGGAGGTGGTTCTGACTACAAAGACCATGACGGTGATTATAAAGATCATGACATCGATTACAAGGATGACGATGACAAGTAA
CHIKV	100	52	92	CCTGTGTACTCGCCTCCGATCAACGTCCGATCGTCCAATCCCGAGTCCGCTGTGGCAGCATGCAATGAGTTCTTAGCTAGAAACTATCCAACTGTCTCAT
	126	56	95.4	CGTGCAGAGCACCGCCGCAACTACCGAAGAGATAGAGGTACACATGCCCCCAGACACCCCTGATCGCACATTAATGTCACAACAGTCCGGCAACGTAAAGATCACAGTCAATGGCCAGACGGTGCG
	100	31	83.4	AGGGACGTAGGAGATGTTATTTTGTTTTTAATATTTCAAAAAAAAAAAAAAAAAAAAAAAAAAAAAAAAAAAAGGCCGGCATGGTCCCAGCCTCCTCGCT

DENV	110	51	92.4	GGCGTGTACGGTGGGAGGTCTATATAAGCAGAGCTCGTTTAGTGAACCGTATGGCTGCGTGAGACACACGTAGCCTACCAGTTTCTTACTGCTCTACTCTGCAAAGCAAG
	100	50	91.2	GGCGTGTACGGTGGGAGGTCTATATAAGCAGAGCTCGTTTAGTGAACCGAGTTGTTAGTCTACGTGGACCGACAAGAACAGTTTCGAATCGGAAGCTTGC
	100	59	94.9	TCACCGATCCAGCCAGCATAGCGGCCAGAGGGTACATCTCAACCCGAGTTGGCATGGGTGAAGCAGCTGCGATCTTCATGACAGCCACTCCCCCAGGATC
	100	53	92.4	TGACAGCCACTCCCCCAGGATCGGTGGAGGCCTTTCCACAGAGCAATGCCGTTATCCAAGATGAGGAAAGAGACATTCCTGAGAGATCATGGAACTCAGG
	100	36	85.5	CCAGAAAATGGAATGGTGCTGTTGAATCAACAGGTTCTAAAAAAAAAAAAAAAAAAAAAAAAAAAAAAAAAAAGGCCGGCATGGTCCCAGCCTCCTCGCT
	100	51	91.6	GGCGTGTACGGTGGGAGGTCTATATAAGCAGAGCTCGTTTAGTGAACCGAGTTGTTAGTCTACGTGGACCGACAAGAACAGTTTCGACTCGGAAGCTTGC
	100	47	90	CTTGATGCCCGCACTTATTCAGATCCCTTAGCACTCAAGGAATTCAAGGACTTTGCGGCCGGTAGAAAGTCAATCGCCCTTGATCTTGTGACAGAAATAG
	100	36	85.5	CCAGAAAATGGAATGGTGCTGTTGAATCAACAGGTTCTAAAAAAAAAAAAAAAAAAAAAAAAAAAAAAAAAAAGGCCGGCATGGTCCCAGCCTCCTCGCT

Table S4. Sanger sequencing data of the region of interest for the generated mutant rSARS-CoV-2, rCHIKV and rDENV1/rDENV3.

	Mutation/ Region	Sequence
SARS-CoV-2	Wuhan(BA.1 S) S	ATGGCAGATTCCAACGGTACTATTACCGTTGAAGAGCTTAAAAAGCTCCTTGAACAATGG
	Wuhan(BA.1 S) M	GGTAACAAACCTTGTAATGGTGTTGCAGGTTTTAATTGTTACTTTCCTTTACGATCATATAGTTTCCGACCCACTTATGGTGTTGGTCAC
	Wuhan(BA.5 S) S	ATGGCAGATTCCAACGGTACTATTACCGTTGAAGAGCTTAAAAAGCTCCTTGAACAATGG
	Wuhan(BA.5 S) M	GGTAACAAACCTTGTAATGGTGTTGCAGGTGTTAATTGTTACTTTCCTTTACGATCATATGGTTTCCAACCCACTTATGGTGTTGGTCAC
	rCOV2-N501Y	TACTTTCCTTTACAATCATATGGTTTCCAACCCACTTATGGTGTTGGTTACCAACCATACAGAGTAGTAGTACTTTCTTTTGAACTTCTACATGCACCAGCAAC<br>TGTTTGT
	rCOV2-N501Y p5	TACTTTCCTTTACAATCATATGGTTTCCAACCCACTTATGGKGTTGGTTACCACCCATACAGAGTAGTAGTACTTTCTTTTGAACTTCTACATGCACCAGCAAC<br>TGTTTGT
	rCOV2-G614D	CCAGGAACAAATACTTCTAACCAGGTTGCTGTTCTTTATCAGGATGTTAACTGCACAGAAGTCCCTGTTGCTATTCATGCAGATCAACTTACTCCTACTTGGC<br>GTGTT
	rCOV2-G614D p5	CCAGGAACAAATACTTCTAACCAGGTTGCTGTTCTTTATCAGGATGTTAACTGCACAGAAGTCCCTGTTGCTATTCATGCAGATCAACTTACTCCTACTTGGC<br>GTGTT
	rCOV2-ΔORF3a	GACTCTGAGCCAGTGCTCAAAGGAGTCAAATTACATTACACATAAGCACAAGCTGATGAGTACGAACTTATGTACTCATTCGTTTCGGAAGAGACAGGTACG<br>TTAATAGTTAATAGCGTACTTCTTTTT
	rCOV2-ORF8-FLAG	ATGAAATTTCTTGTTTTCTTAGGAATCATCACAACTGTAGCTGCATTTCACCAAGAATGTAGTTTACAGTCATGTACTCAACATCAACCATATGTAGTTGATGA<br>CCCGTGTCCTATTCACTTCTATTCTAAATGGTATATTAGAGTAGGAGCTAGAAAATCAGCACCTTTAATTGAATTGTGCGTGGATGAGGCTGGTTCTAAATCA<br>CCCATTCAGTACATCGATATCGGTAATTATACAGTTTCCTGTTTACCTTTTACAATTAATTGCCAGGAACCTAAATTGGGTAGTCTTGTAGTGCGTTGTTCGTT<br>CTATGAAGACTTTTTAGAGTATCATGACGTTCGTGTTGTTTTAGATTTCATCGGCGGAGGTGGTTCTGACTACAAAGACCATGACGGTGATTATAAAGATCAT<br>GACATCGATTACAAGGATGACGATGACAAGTAAACGAACAAACTAAAATGTCT
	rCoV2-link-Omicron BA.1(BA.5 S) S	GGTAACAAACCTTGTAATGGTGTTGCAGGTGTTAATTGTTACTTTCCTTTACAATCATATGGTTTCCGACCCACTTATGGTGTTGGTCAC
	rCoV2-link-Omicron BA.1(BA.5 S) M	ATGGCAGGTTCCAACGGTACTATTACCGTTGAAGAGCTTAAAAAGCTCCTTGAAGAATGG
	rCoV2-link-Omicron BA.5(BA.1 S) S	GGTAACAAACCTTGTAATGGTGTTGCAGGTTTTAATTGTTACTTTCCTTTACGATCATATAGTTTCCGACCCACTTATGGTGTTGGTCAC
	rCoV2-link-Omicron BA.5(BA.1 S) M	ATGGCAAATTCCAACGGTAYTATTACCGTTGAAGAGCTTAAAAAGCTCCTTGAAGAATGG
	rCoV2-link-Omicron BA.5(Wuhan S) S	GGTAGCACACCTTGTAATGGTGTTGAAGGTTTTAATTGTTACTTTCCTTTACATTCATATGGTTTCCAACCCACTAATGGTGTTGGTTAC
	rCoV2-link-Omicron BA.5(Wuhan S) M	ATGGCAAATTCCAACGGTACTATTACCGTTGAAGAGCTTAAAAAGCTCCTTGAAGAATGG
	BA.5 Δ3	ACGACTCTGAGCCAGTGCTCAAAGGAGTCAAATTACATTACACATAAGCACAAGCTGATGAGTACGAACTTATGTACTCATTCGTTTCGGAAGAGATAGGTA<br>CGTTAATA
	BA.5 Δ3 M	ATGGCAAATTCCAACGGTACTATTACCGTTGAAGAGCTTAAAAAGCTCCTTGAAGAATGG
	XBB Δ3	ACGACTCTGAGCCAGTGCTCAAAGGAGTCAAATTACATTACACATAAGCACAAGCTGATGAGTACGAACTTATGTACTCATTCGTTTCGGAAGAGATAGGTG<br>CGTTAATAAAT
	XBB Δ3 M	ATGGCAGATTCCAACGGTACTATTACCGTTGAAGAGCTTAAAAAGCTCCTTGAAGAATGG
CHIKV	rCHIKV	GCGCCTGTGTACTCGCCTCCGATCAACGTCCGATTGTCCAATCCCGAGTCCGCTGTGGCAGCATGCAATGAGTTCTTAGCTAGAAACTATCCAACTGTCTC<br>ATCATACCAAATTACCGACGAGTATGATGCATATCTAGACATGGTGGACGGGTCGGAGAGTTGCCTGGACCGAGCGACATTCAATCCGTCAAAACTCAGGA<br>GCTACCCGAAACAGCACGCTTACCACGCGCCCTCCATCAGAAGCGCTG

	CHIKV ctr	GCGCCTGTGTACTCGCCTCCGATCAACGTCCGATTGTCCAATCCCGAGTCCGCAGTGGCAGCATGCAATGAGTTCTTAGCTAGAAACTATCCAACTGTCTC ATCATACCAAATTACCGACGAGTATGATGCATATCTAGACATGGTGGACGGGTCGGAGAGTTGCCTGGACCGAGCGACATTCAATCCGTCAAAACTCAGGA GCTACCCGAAACAGCACGCTTACCACGCGCCCTCCATCAGAAGCGCTG
DENV	rDENV1 A	TACCGATCCAGCTAGCATAGCGGCCAGAGGGTACATCTCAACCCGAGTGGGCATGGGTGAAGCAGCTGCGATCTTTATGACAGCCACTCCCCCAGGATCG GTGGAGGCCTTTCCACAGAGCAATGCCGTTATCCAAGATGAGGAAAGAGACATTCCTGAGAGATCATGGAACTCAGGCTACGACTGGATCACTGACTTTCC AGGTAAAACAGTCTGGTTTGTTCCAAGCATCAAG
	DENV1 ctr	TACCGATCCAGCTAGCATAGCGGCCAGAGGGTACATCTCAACCCGAGTGGGCATGGGTGAAGCAGCTGCGATCTTTATGACAGCCACTCCCCCAGGATCG GTGGAGGCCTTTCCACAGAGCAATGCAGTCATCCAAGATGAGGAAAGAGACATTCCTGAGAGATCATGGAACTCAGGCTACGACTGGATCACTGACTTTCC AGGTAAAACAGTCTGGTTTGTTCCAAGCATCAAG
	rDENV1 B	TATGGATGAAGCACATTTTACCGATCCAGCCAGCATAGCGGCCAGAGGGTACATCTCAACCCGAGTTGGCATGGGTGAAGCAGCTGCGATCTTTATGACAG CCACTCCCCCAGGATCGGTGGAGGCCTTTCCACAGAGCAATGCAGTCATCCAAGATGAGGAAAGAGACATTCCTGAGAGATCATGGAACTCAGGCTACGA CTGGATCACTGACTTTCCAGGTAAAACAGTCTGGTTTGTTCCAAGCATCAAG
	rDENV3	GTAGCATCAGAAGGGATCAAATATACAGATAGGAAATGGTGCTTTGATGGACAGCGCAACAACCAAATTTTAGAGGAAAACATGGACGTGGAAATCTGGACA AAGGAAGGAGAAAAGAAAAAATTAAGACCTAGGTGGCTTGATGCCCGCACTTATTCAGATCCCTTAGCACTCAAGGAATTCAAGGACTTTGCGGCCGGTAG AAAGTCAATCGCCCTTGATCTTGTGACAGAAATAGGAAGAGTGCCTTCACACCTAGCCCACAGAACGAGAAACGCTCTGGACAATT
	DENV3 ctr	GTAGCATCAGAAGGGATCAAATATACAGATAGGAAATGGTGCTTTGATGGACAGCGCAACAACCAAATTTTAGAGGAAAACATGGACGTGGAAATCTGGACA AAGGAAGGAGAAAAGAAAAAATTAAGACCTAGGTGGCTTGATGCCCGCACTTATTCAGATCCCTTAGCACTCAAGGAATTCAAGGACTTTGCGGCTGGTAGA AAGTCAATCGCCCTTGATCTTGTGACAGAAATAGGAAGAGTGCCTTCACACCTAGCCCACAGAACGAGAAACGCTCTGGACAATT

Table S5. Primer tables.

	Fragment	Primer	Sequence 5' to 3'	Amplicon Size
SARS-CoV-2	A1	F CMV	CGATGTACGGGCCAGATATACG	4694
		R A1-A2	GGCAGAATCTGGATGAAGATTGCC	
	A2	F A2-A1	TGTGTTGAAGAAGTTACAACAACTCTG	4672
		R A-B	GTGTTATTAAATAGAAAATAGCAGCAACAAAAAGGAACACAAGTGT AACTTTAATTAACTGCTTCAACC	
	B1	F B-A	GCACTTAAGGGTGGTAAAATTGTTAATAATTGGTTGAAGCAGTTAAT TAAAGTTACACTTGTGTTCC	3292
		R B1-B2	CTACTTTGATACAAGGTTTGCCACC	
	B2	F B2-B1	CACAGGGACTACTCCCACCC	3415
		R B-C	AAACTGTCTATTGGTCATAGTACTACAGATAGAGACACCAGCTACG GTGCGAGCTCTATTCTTTGCAC	
	C1	F C-B	TATAACTCAAATGAATCTTAAGTATGCCATTAGTGCAAAGAATAGAG CTCGCACCGTAGCTGGTG	3048
		R C1-C2	GTAGGATGTAACCCAGTGATTACC	
	C2	F C2-C1	TTTACAAGTCTTGAAATTCCACGTAGG	3006
		R C-D	ATCACCAATCAAAGTTGAATCTGCATCAGAGACAAAGTCATTAAGAT CTGAGTCGACAAGCAGCG	
	D1	F D-C	TACAGCTGTTTTAAGACAGTGGTTGCCTACGGGTACGCTGCTTGTC GACTCAGATCTTAATGACTTTGTC	4622
		R D1-D2	CGAAAGGGAGTGAGGCTTGTATC	
	D2	F D2-D1	GGAACTGTAACTTTGAAGCAAGGTG	4707
		R SV40	GCGGCCGCCAGACATGATAAG	
	A	F CMV	CGATGTACGGGCCAGATATACG	9264
		R A-B	GTGTTATTAAATAGAAAATAGCAGCAACAAAAAGGAACACAAGTGT AACTTTAATTAACTGCTTCAACC	
	B	F B-A	GCACTTAAGGGTGGTAAAATTGTTAATAATTGGTTGAAGCAGTTAAT TAAAGTTACACTTGTGTTCC	6614
		R B-C	AAACTGTCTATTGGTCATAGTACTACAGATAGAGACACCAGCTACG GTGCGAGCTCTATTCTTTGCAC	
	C	F C-B	TATAACTCAAATGAATCTTAAGTATGCCATTAGTGCAAAGAATAGAG CTCGCACCGTAGCTGGTG	5947
		R C-D	ATCACCAATCAAAGTTGAATCTGCATCAGAGACAAAGTCATTAAGAT CTGAGTCGACAAGCAGCG	
	D	F D-C	TACAGCTGTTTTAAGACAGTGGTTGCCTACGGGTACGCTGCTTGTC GACTCAGATCTTAATGACTTTGTC	9229
		R SV40	GCGGCCGCCAGACATGATAAG	
	D1 614	F D-C		2535

		R G614D	GAATAGCAACAGGGACTTCTGTGCAGTTAACATCCTGATAAAGAAC AGCAACCTGGTTAGAAGTATTTGTTCC	
	D2 614	F G614D	AACACCAGGAACAAATACTTCTAACCAGGTTGCTGTTCTTTATCAG GATGTTAACTGCACAGAAGTCCCTGTTGCTATTC	6774
		R SV40		
	D1 501	F D-C		2212
		R N501Y	AGTTCAAAAGAAAGTACTACTACTCTGTATGGTTGGTAACCAACAC CATAAGTGGGTTGGAAACCATATG	
	D2 501	F N501Y	AAGGTTTTAATTGTTACTTTCCTTTACAATCATATGGTTTCCAACCCA CTTATGGTGTTGGTTACCAACCATAC	7117
		R SV40		
	D1 Δorf3a	F D-C		4495
		R Δorf3a	CCTGTCTCTTCCGAAACGAATGAGTACATAAGTTCGTACTCATCAG CTTGTGCTTATGTGTAATGTAATTTGACTCC	
	D2 Δorf3a	F Δorf3a	ACGACTCTGAGCCAGTGCTCAAAGGAGTCAAATTACATTACACATA AGCACAAGCTGATGAGTACGAACTTATGTACTC	3912
		R SV40		
	D1 orf8-3xFLAG	F D-C		7356
		R orf8-3xFLAG	TTACTTGTCATCGTCATCCTTGTAATCGATGTCATGATCTTTATAATC ACCGTCATGGTCTTTGTAGTCAGAACCACCTCCGCCGATGAAATCT AAAACAACACG	
	D2 orf8-3xFLAG	F orf8-3xFLAG	GGCGGAGGTGGTTCTGACTACAAAGACCATGACGGTGATTATAAA GATCATGACATCGATTACAAGGATGACGATGACAAGTAAACGAACA AACTAAAATGTCTG	1873
		R SV40		
	linker	F linker	AGGCCACGCGGAGTACGATCGAGTGTACAGTGAACAATGCTAGGG AGAGCTGCC<br>or<br>TGCTAGGGAGAGCTGCC (equal performance)	1106
		R linker	CAGCCGAGTGACAGCCACAC	
	A1-linker	F A-linker	GTTTAGTGAACCGTATATTAGGTTTATACCTTCCCAGGTAACAAACC	4083
		R A1-A2		
	D2-linker	F D2-D1		4451
		R D-linker	TTTGTCATTCTCCTAAGAAGCTATTAAAATCACATG	
	D1 500	F D-C		500
		R 500	TCATGTCAAATAAAGAATAGGAAGACAACTG	
	D2 500	F 500	CCACGCGAACAAATAGATGGTTATG	8829
		R SV40		

CHIKV	fr1	F fr1-linker CHIKV	GGCGTGTACGGTGGGAGGTCTATATAAGCAGAGCTCGTTTAGTGAA CCGTATGGCTGCGTGAGACACACG	6128
		R fr1-fr2 CHIKV	ATG AGA CAG TTG GAT AGT TTC TAG CTA AGA ACT CAT TGC ATG CTG CCA CAG CGG ACT CGG GAT TGG AC	
	fr2	F fr2_fr1 CHIKV	CCT GTG TAC TCG CCT CCG ATC AAC GTC CGA TCG TCC AAT CCC GAG TCC GCT GTG GCA GCA TGC AAT GAG	3157
		R fr2-fr3 CHIKV	CGCACCGTCTGGCCATTGACTGTGATCTTTACGTTGCCGGACTGTT GTGACATTAATGTGCGATCAGGGGTG	
	fr3	F fr3-fr2 CHIKV	CGTGCAGAGCACCGCCGCAACTACCGAAGAGATAGAGGTACACAT GCCCCCAGACACCCCTGATCGCAC	2816
		R fr3-linker CHIKV	AGCGAGGAGGCTGGGACCATGCCGGCCTTTTTTTTTTTTTTTTTTTT TTTTTTTTTTTTTTTTGAAATATTAAAAACAAAATAACATCTCC	
	linker CHIKV	F linker-fr3 CHIKV	AGGGACGTAGGAGATGTTATTTTGTTTTTAATATTTCAAAAAAAAAAA AAAAAAAAAAAAAAAAAAAAAAAAGGCCGGCATGGTCCCAG	1009
		R linker-fr1 CHIKV	CTTGCTTTGCAGAGTAGAGCAGTAAGAAACTGGTAGGCTACGTGTG TCTCACGCAGCCATACGGTTCACTAAACGAGC	

DENV	linker DENV1	F linker-fr2_DENV1	CCA GAA AAT GGA ATG GTG CTG TTG AAT CAA CAG GTT CTA AAA AAA AAA AAA AAA AAA AAA AAA AAA AAA AAA AGG CCG GCA TGG TCC CAG	989
		R linker-fr1_DENV1	GCA AGC TTC CGA TTC GAA ACT GTT CTT GTC GGT CCA CGT AGA CTA ACA ACT CGG TTC ACT AAA CGA GC	
	fr1 mA	F fr1-linker_DENV1	GGC GTG TAC GGT GGG AGG TCT ATA TAA GCA GAG CTC GTT TAG TGA ACC GAG TTG TTA GTC TAC GTG GAC C	5612
		R fr1-fr2 DENV1 A	CCT GAG TTC CAT GAT CTC TCA GGA ATG TCT CTT TCC TCA TCT TGG ATA ACG GCA TTG CTC TGT GGA AAG GCC TCC AC	
	fr2 mA	F fr2-fr1 DENV1 A	TGA CAG CCA CTC CCC CAG GAT CGG TGG AGG CCT TTC CAC AGA GCA ATG CCG TTA TCC AAG ATG AGG AAA GAG	5335
		R fr2-linker_DENV1	AGCGAGGAGGCTGGGACCATGCCGGCCTTTTTTTTTTTTTTTTTTTT TTTTTTTTTTTTTTTTAGAACCTGTTGATTCAACAGCACCATTCCATTTT CTGGCGTTCTGTGCCTGGAATGAT	
	fr1 mB	F fr1-linker_DENV1	GGC GTG TAC GGT GGG AGG TCT ATA TAA GCA GAG CTC GTT TAG TGA ACC GAG TTG TTA GTC TAC GTG GAC C	5534
		R fr1-fr2 DENV1 B	GAT CCT GGG GGA GTG GCT GTC ATG AAG ATC GCA GCT GCT TCA CCC ATG CCA ACT CGG GTT GAG ATG TAC CCT C	
	fr2 mB	F fr2-fr1 DENV1 B	TCA CCG ATC CAG CCA GCA TAG CGG CCA GAG GGT ACA TCT CAA CCC GAG TTG GCA TGG GTG AAG CAG CTG C	5413
		R fr2-linker_DENV1	AGCGAGGAGGCTGGGACCATGCCGGCCTTTTTTTTTTTTTTTTTTTT TTTTTTTTTTTTTTTTagaacctgttgattcaacagcaccattccattttctggcgttctgtgcctg gaatgat	
	linker DENV3	F linker-fr2 DENV3	CCA GAA AAT GGA ATG GTG CTG TTG AAT CAA CAG GTT CTA AAA AAA AAA AAA AAA AAA AAA AAA AAA AAA AAA AGG CCG GCA TGG TCC CAG	989
		R linker-fr1 DENV3	GCA AGC TTC CGA GTC GAA ACT GTT CTT GTC GGT CCA CGT AGA CTA ACA ACT CGG TTC ACT AAA CGA GC	
	fr1	F fr1-linker DENV3	GGC GTG TAC GGT GGG AGG TCT ATA TAA GCA GAG CTC GTT TAG TGA ACC GAG TTG TTA GTC TAC GTG GAC C	6450

		R fr1-fr2 DENV3	CTA TTT CTG TCA CAA GAT CAA GGG CGA TTG ACT TTC TAC CGG CCG CAA AGT CCT TGA ATT CCT TGA GTG CTA AGG GAT CTG AAT AAG	
	fr2	F fr2-fr1 DENV3	CTT GAT GCC CGC ACT TAT TCA GAT CCC TTA GCA CTC AAG GAA TTC AAG GAC TTT GCG GCC GGT AGA AAG TCA ATC GCC CTT GAT CTT GTG AC	4468
		R fr2-linker_DENV3	AGCGAGGAGGCTGGGACCATGCCGGCCTTTTTTTTTTTTTTTTTTTT TTTTTTTTTTTTTTTagaacctgttgattcaacagcaccattccattttctggcgttctgtgcctg gaatgat	

Table S6. PCR settings for individual fragments.

	Fragment	Cycling Conditions	Elongation
SARS-CoV-2	A	5 cycles at 57 °C	4 min 40 s
		20 cycles at 65 °C	
	B	5 cycles at 47 °C	3 min 20 s
		20 cycles at 65 °C	
	C	5 cycles at 54 °C	3 min
		20 cycles at 65 °C	
	D	5 cycles at 50 °C	4 min 20 s
		20 cycles at 65 °C	
	A1	5 cycles at 52 °C	2 min 30 s
		20 cycles at 64 °C	
	A2	5 cycles at 52 °C	2 min 30 s
		20 cycles at 64 °C	
	B1	5 cycles at 48 °C	1 min 45 s
		20 cycles at 63 °C	
	B2	5 cycles at 48 °C	1 min 45 s
		20 cycles at 63 °C	
	C1	5 cycles at 48 °C	1 min 45 s
		20 cycles at 63 °C	
	C2	25 cycles at 61 °C	1 min 45 s
	D1	25 cycles at 63 °C	2 min 30 s
	D2	5 cycles at 52 °C	2 min 30 s
		20 cycles at 64 °C	
	linker	25 cycles at 58 °C	35 s
CHIKV	linker CHIKV	5 cycles at 54 °C	30 s
		20 cycles at 64 °C	
	fr1 CHIKV	25 cycles at 64 °C	3 min 10 s
	fr2 CHIKV	25 cycles at 62 °C	1 min 40 s
	fr3 CHIKV	25 cycles at 61 °C	1 min 30 s

DENV	linker DENV1	5 cycles at 52 °C	30 s
		20 cycles at 62 °C	
	fr1 DENV1 A	5 cycles at 55 °C	2 min 50 s
		20 cycles at 62 °C	
	fr2 DENV1 A	5 cycles at 50 °C	2 min 50 s
		20 cycles at 62 °C	
	fr1 DENV1 B	5 cycles at 55 °C	2 min 50 s
		20 cycles at 62 °C	
	fr1 DENV1 B	5 cycles at 50 °C	2 min 50 s
		20 cycles at 62 °C	
	linker DENV3	5 cycles at 52 °C	30 s
		20 cycles at 62 °C	
	fr1 DENV3	5 cycles at 50 °C	3 min 20 s
		20 cycles at 62 °C	
	fr2 DENV3	5 cycles at 50 °C	2 min 20 s
		20 cycles at 62 °C	
