# Supplementary material for: Rapid cloning-free mutagenesis of new SARS-CoV-2 variants using a novel reverse genetics platform
Source: bioRxiv. 2023 May 30:2023.05.11.540343. Preprint. [Version 3] doi: 10.1101/2023.05.11.540343 (PMC10245781; doi:10.1101/2023.05.11.540343)
Supplement: Supplementary file 1 [file media-1.pdf]

Supplementary Materials for:

Rapid cloning-free mutagenesis of new SARS-CoV-2 variants using a novel reverse genetics platform

Enja Kipfer, David Hauser, Martin J. Lett, Fabian Otte, Lorena Urda, Yuepeng Zhang, Christopher M. R. Lang, Mohamed Chami, Christian Mittelholzer, Thomas Klimkait

Figure S1. Clonal virus populations and reconstitution efficiency

A Schematic representation of the work flow to generate clonal virus populations. Right after transfection, cells were diluted to less than 0.5 virus-producing cells/well in 96-well plates. 7 days post transfection, supernatant was transferred onto Vero E6-TMPRSS2 cells. Plates were observed until first CPE was visible.

B Clonal virus populations arising from a single virus-producing cell were identified after supernatant transfer onto Vero E6-TMPRSS2 cells. CPE of infectious virus was assessed by microscopy and virus was collected for further analysis. Thereafter, plates were fixed and stained for the fast enumeration of positive wells (in light blue).

Figure S2. CLEVER primer design for direct mutagenesis

Shown are the different approaches of primer design in order to guarantee a 100 bp overlap between the neighboring fragments. Primers can either be separated in distance to ensure homology regions or additional nucleotides must be added to the 5' end of the primer. Small nucleotide changes, deletions or insertions can directly be introduced by adjusting the primer annealing sites and the homology regions, as depicted.

Figure S3. Cloning-free rescue of chimeric virus

A In addition to the chimeric viruses described in Figure 3, the genetic background (region outside of S) was replaced by the sequence of Omicron BA.1 or Omicron BA.5 and combined with a heterologous S sequence of Wuhan, Omicron BA.1 or BA.5, respectively.

B Infectious chimeric virus was rescued and assessed via CPE formation on Vero E6 cells. Scale bar represents 100 µm.

C The fragments were directly amplified by one-step RT-PCR from viral RNA of Wuhan, Omicron BA.1 and Omicron BA.5. Eight fragments were amplified to prove high flexibility in exchanging fragments.

D Schema for the rapid distinguishment between Wuhan, Omicron BA.1, BA.5 and XBB.1.5 variants. Indicated regions (S or M) were Sanger sequenced to discriminate variants or confirm chimeric viruses. Amino acids for the clear identification of the S gene variant and/or the background (within M) are highlighted in yellow.

Table S1. Infectivity assessment of recombinant virus on Vero E6 cells.

Vero E6 cells were infected with the indicated recombinant virus and pictures were taken 3 days post infection. Note that virus was not titrated and development of CPE is not quantitative. Scale bar represents 100 µm.

Table S2. Genomic characterization of recombinant SARS-CoV-2 virus based on NGS data (10 % cut-off).

Table S3. Homology regions successfully used for virus rescue.

Length, GC content as well as the hypothetical annealing temperature (according to OligoCalc, salt-adjusted) are listed. Note that homology regions were chosen independently from GC content or annealing temperature and values are only listed for completion.

Table S4. Sanger sequencing data of the region of interest for the generated mutant recombinant SARS-CoV-2 viruses.

Table S5. Primers used for the generation of recombinant SARS-CoV-2 virus.

Table S6. PCR settings for individual fragments.

Figure S1: Clonal virus populations and reconstitution efficiency

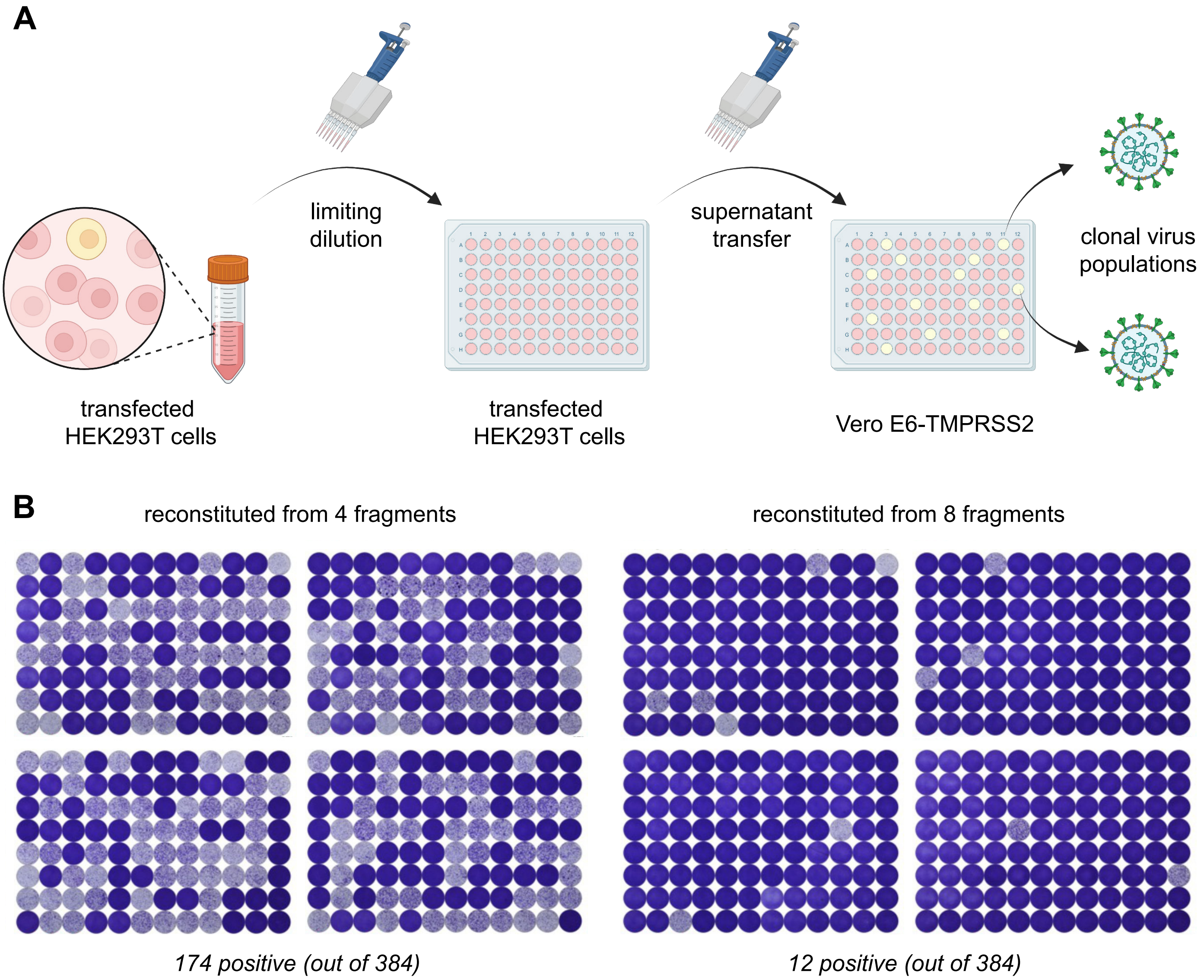

Figure S2: CLEVER primer design for direct mutagenesis

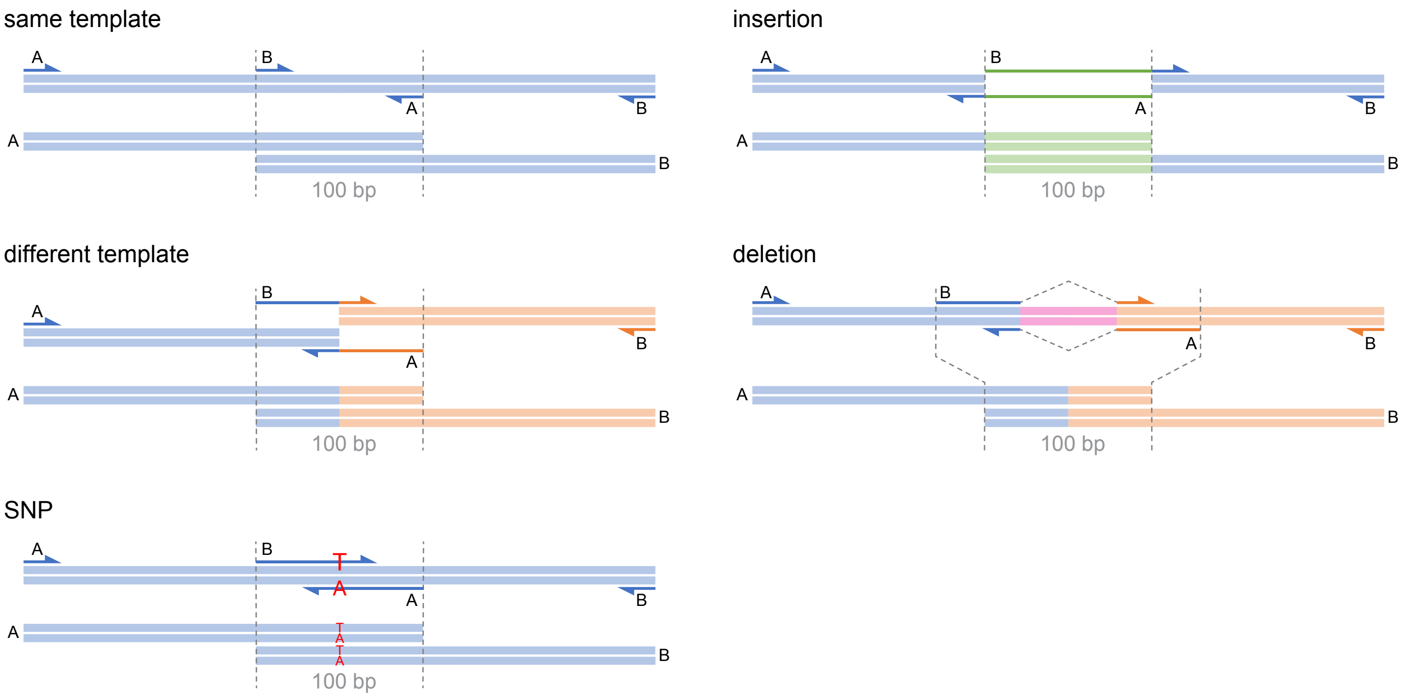

Figure S3: Cloning-free rescue of chimeric virus

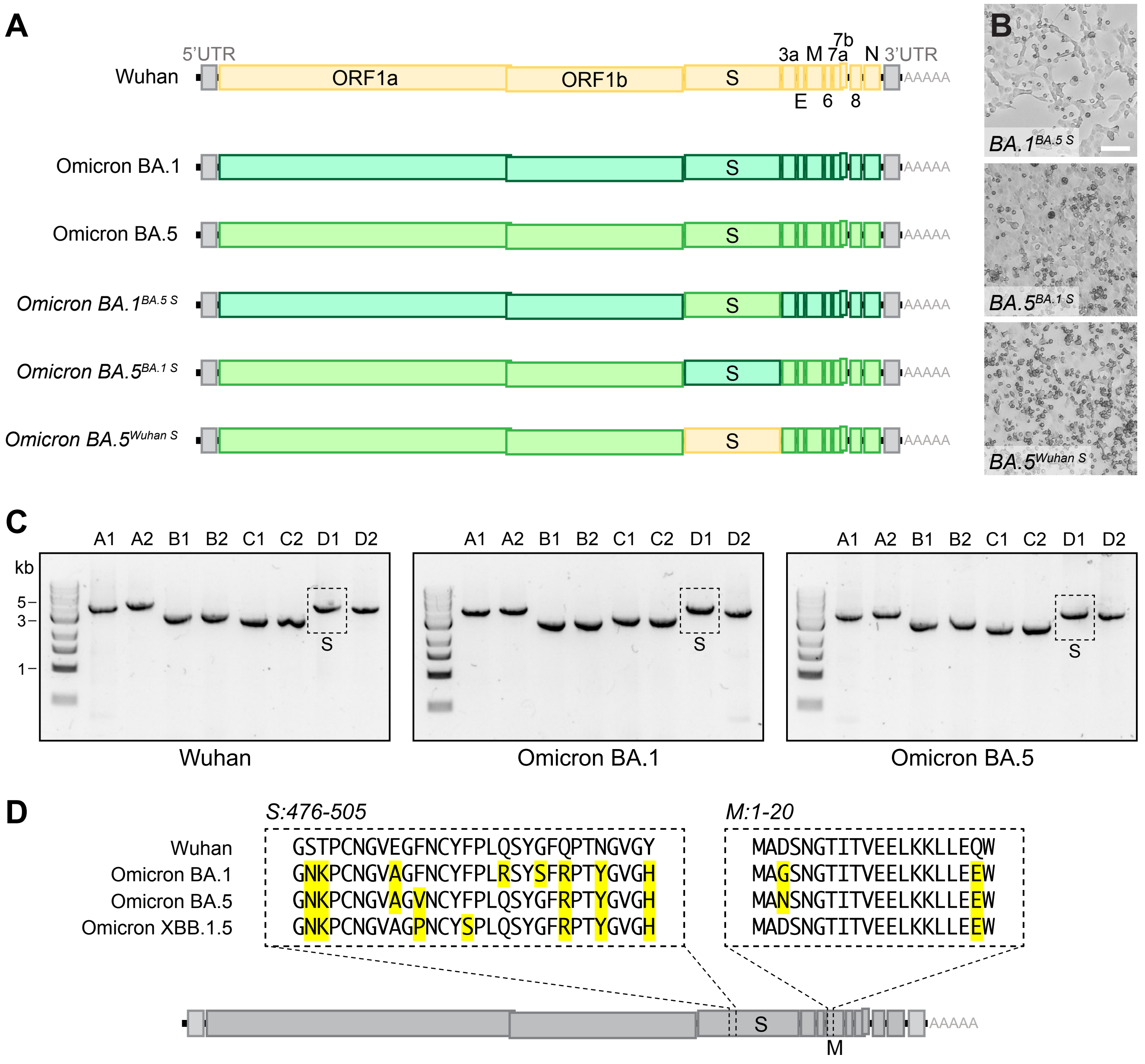

Table S1. Infectivity assessment of recombinant virus on Vero E6 cells.

Number of fragments	Transfection method	Name	Cell line transfected	3 days post infection on Vero E6	Description
-	-	Vero E6 negative control	-	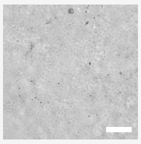	Uninfected Vero E6 cells.
8	electroporation	rCOV2-8fr	HEK293T	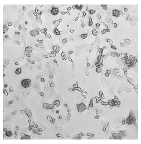	Rescue from eight fragments.
4	electroporation	rCOV2-4fr	HEK293T	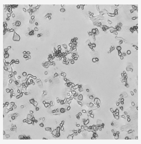	Rescue from four fragments.
4	electroporation	rCOV2-293	HEK293	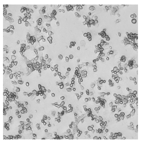	Rescue from HEK293.
4	electroporation	rCOV2-CHO	CHO	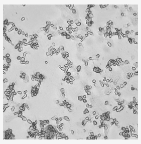	Rescue from CHO.
4	electroporation	rCOV2-BHK	BHK-21	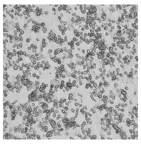	Rescue from BHK-21.
4	electroporation	rCOV2-A549	A549-ACE2	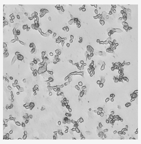	Rescue from A549-ACE2.
4	jetPRIME	rCOV2-jP	HEK293T	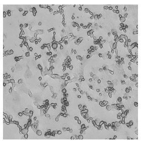	Rescue after transfection with jetPRIME.
4	Lipofectamine-3000	rCOV2-3000	HEK293T	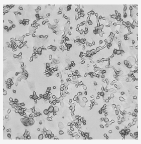	Rescue after transfection with Lipofectamine-3000.
4	Lipofectamine-LTX	rCOV2-LTX	HEK293T	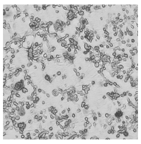	Rescue after transfection with Lipofectamine-LTX.

5	electroporation	rCOV2-500bp	HEK293T	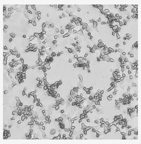	Rescue including one fragment to be only 500 bp in length.
5	electroporation	Wuhan$^{\text{BA.1 S}}$	HEK293T	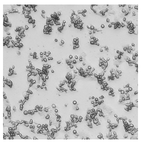	Chimeric virus, Omicron BA.1 S gene in the background of Wuhan.
5	electroporation	Wuhan$^{\text{BA.5 S}}$	HEK293T	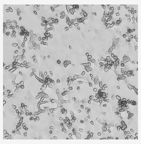	Chimeric virus, Omicron BA.5 S gene in the background of Wuhan.
5	electroporation	rCOV2-N501Y	HEK293T	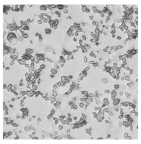	Direct mutagenesis, incorporation of N501Y substitution in the S gene.
5	electroporation	rCOV2-G614D	HEK293T	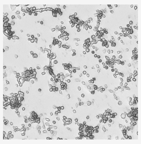	Direct mutagenesis, incorporation of G614D substitution in the S gene.
5	electroporation	rCOV2-ΔORF3a	HEK293T	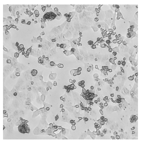	Direct mutagenesis, deletion of ORF3a.
5	electroporation	rCOV2-ORF8-FLAG	HEK293T	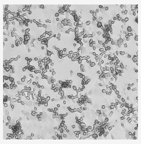	Direct mutagenesis, incorporation of a 3x-FLAG tag at the C-terminal of ORF8.
9	electroporation	rCOV2-link-Wuhan	HEK293T	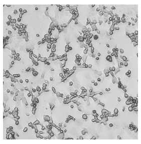	Rescue from RNA directly using the linker fragment.
9	electroporation	rCoV2-link-Omicron BA.5$^{\text{BA.1 S}}$	HEK293T	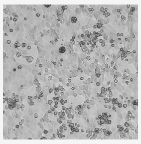	Rescue of chimeric virus from RNA directly using the linker fragment. Omicron BA.1 S gene in the background of Omicron BA.5.
9	electroporation	rCoV2-link-Omicron BA.1$^{\text{BA.5 S}}$	HEK293T	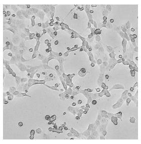	Rescue of chimeric virus from RNA directly using the linker fragment. Omicron BA.5 S gene in the background of Omicron BA.1.
9	electroporation	rCoV2-link-Omicron BA.5$^{\text{Wuhan S}}$	HEK293T	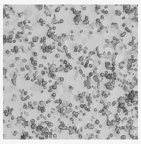	Rescue of chimeric virus from RNA directly using the linker fragment. Wuhan S gene in the background of Omicron BA.5.

Table S2. Genomic characterization of recombinant SARS-CoV-2 virus based on NGS data (10 % cut-off).

ID	position							"N" reads*	origin of template
	13551	21003*	21621*	21638*	25768*	28540*	28967*		
1		K	M	Y	M				T15324C
2			M	Y	M			5979, 9628, 9629, 20231, 20525, 20526	T15324Y
3			M	Y	M	M	M		T15324Y
4			M	Y	M	M			T15324Y
5			M	Y	M	M		5979, 5980, 9628, 9629, 20525, 21002, 21003	
6		K	M	Y	M	M	M	5979, 5980, 9628, 9629, 20525, 20526	T15324Y
7	C to T (13 %)		M	Y	M	M		9628, 9629	T15324Y
8		K	M	Y	M	M		5979, 9628, 9629, 20231, 20232, 20526	T15324Y
	orf1ab		*S*		*orf3a*	*N*			

* Ambiguities or "N" stretches occuring as clusters were not considered as a randomly introduced mutation during the PCR-amplification step but rather as technical artefact or culture adaptation of the virus.

ID	position							
	*29***	*30***	*79*	*259*	*263*	*264*	550	12464
clonal 1								
clonal 2	*A to G*	*A to G*	*A to G*	*A to G*	*A to G*	*A to G*	A to G	
clonal 3								
clonal 4								
clonal 5								G to T
	5'UTR						*orf1ab*	

** low coverage (20 reads)

ID	position														
	3561	5834	6067	18750	18897	19097	20429	20763	20855	20881	21137	21777	25101	27787	28720
RNA clonal 1		T to C		T to C							A to G		A to G	T del	C to T
RNA clonal 2							C to T	A to G	G to C	G to A					
RNA clonal 3	T to C		T to G		T to C	C to T						G to A			
	orf1ab											*S*		*orf7b*	*N*

Table S3. Homology regions successfully used for virus rescue. Length, GC content as well as the hypothetical annealing temperature (according to OligoCalc, salt-adjusted) are listed. Note that homology regions were chosen independently from GC content or annealing temperature and values are only listed for completion.

Length (bp)	G/C (%)	Annealing (°C)	Sequence
100	30	83	GCACTTAAGGGTGGTAAAATTGTTAATAATTGGTTGAAGCAGTTAATTAAAGTTACACTTGTGTTCCTTTTTGTTGCTGCTATTTTCTATTTAATAACAC
100	38	86.3	TATAACTCAAATGAATCTTAAGTATGCCATTAGTGCAAAGAATAGAGCTCGCACCGTAGCTGGTGTCTCTATCTGTAGTACTATGACCAATAGACAGTTT
100	43	88.3	TACAGCTGTTTTAAGACAGTGGTTGCCTACGGGTACGCTGCTTGTCGACTCAGATCTTAATGACTTTGTCTCTGATGCAGATTCAACTTTGATTGGTGAT
102	35	85.2	TGTGTTGAAGAAGTTACAACAACTCTGGAAGAAACTAAGTTCCTCACAGAAAACTTGTTACTTTATATTGACATTAATGGCAATCTTCATCCAGATTCTGCC
93	43	87.7	CACAGGGACTACTCCCACCCAAGAATAGCATAGATGCCTTCAAACTCAACATTAAATTGTTGGGTGTTGGTGGCAAACCTTGTATCAAAGTAG
107	36	86	TTTACAAGTCTTGAAATTCCACGTAGGAATGTGGCAACTTTACAAGCTGAAAATGTAACAGGACTCTTTAAAGATTGTAGTAAGGTAATCACTGGGTTACATCCTAC
100	45	89.2	CAGTCAGCACCTCATGGTGTAGTCTTCTTGCATGTGACTTATGTCCCTGCACAAGAAAAGAACTTCACAACTGCTCCTGCCATTTGTCATGATGGAAAAG
100	46	89.6	GGAACTGTAACTTTGAAGCAAGGTGAAATCAAGGATGCTACTCCTTCAGATTTTGTTCGCGCTACTGCAACGATACCGATACAAGCCTCACTCCCTTTCG
100	34	84.6	CCACGCGAACAAATAGATGGTTATGTCATGCATGCAAATTACATATTTTGGAGGAATACAAATCCAATTCAGTTGTCTTCCTATTCTTTATTTGACATGA
102	37	86	TGCTAGGGAGAGCTGCCTATATGGAAGAGCCCTAATGTGTAAAATTAATTTTAGTAGTGCTATCCCCATGTGATTTTAATAGCTTCTTAGGAGAATGACAAA
119	40	88.4	GTTTAGTGAACCGTATATTAGGTTTATACCTTCCCAGGTAACAAACCAACCAACTTTCGATCTCTTGTAGATCTGTTCTCTAAACGAACTTTAAAATCTGTGTGGCTGTCACTCGGCTG
100	34	84.6	AAGGTTTTAATTGTTACTTTCCTTTACAATCATATGGTTTCCAACCCACTAATGGTGTTGGTTACCAACCATACAGAGTAGTAGTACTTTCTTTTGAACT
80	43	86.3	AACACCAGGAACAAATACTTCTAACCAGGTTGCTGTTCTTTATCAGGGTGTTAACTGCACAGAAGTCCCTGTTGCTATTC
100	43	88.3	ACGACTCTGAGCCAGTGCTCAAAGGAGTCAAATTACATTACACATAAGCACAAGCTGATGAGTACGAACTTATGTACTCATTCGTTTCGGAAGAGACAGG
84	43	86.8	GGCGGAGGTGGTTCTGACTACAAAGACCATGACGGTGATTATAAAGATCATGACATCGATTACAAGGATGACGATGACAAGTAA

Table S4. Sanger sequencing data of the region of interest for the generated mutant recombinant SARS-CoV-2 viruses.

Mutation/Region	Sequence
Wuhan(BA.1 S) S	ATGGCAGATTCCAACGGTACTATTACCGTTGAAGAGCTTAAAAAGCTCCTTGAACAATGG
Wuhan(BA.1 S) M	GGTAACAAACCTTGTAATGGTGTTGCAGGTTTTAATTGTTACTTTCCTTTACGATCATATAGTTTCCGACCCACTTATGGTGTTGGTCAC
Wuhan(BA.5 S) S	ATGGCAGATTCCAACGGTACTATTACCGTTGAAGAGCTTAAAAAGCTCCTTGAACAATGG
Wuhan(BA.5 S) M	GGTAACAAACCTTGTAATGGTGTTGCAGGTGTTAATTGTTACTTTCCTTTACGATCATATGGTTTCCAACCCACTTATGGTGTTGGTCAC
rCOV2-N501Y	TACTTTCCTTTACAATCATATGGTTTCCAACCCACTTATGGTGTTGGTTACCAACCATACAGAGTAGTAGTACTTTCTTTTGAACTTCTACATGCACCAGCAACTGTTTGT
rCOV2-N501Y p5	TACTTTCCTTTACAATCATATGGTTTCCAACCCACTTATGGKGTTGGTTACCACCCATACAGAGTAGTAGTACTTTCTTTTGAACTTCTACATGCACCAGCAACTGTTTGT
rCOV2-G614D	CCAGGAACAAATACTTCTAACCAGGTTGCTGTTCTTTATCAGGATGTTAACTGCACAGAAGTCCCTGTTGCTATTCATGCAGATCAACTTACTCCTACTTGGCGTGTT
rCOV2-G614D p5	CCAGGAACAAATACTTCTAACCAGGTTGCTGTTCTTTATCAGGATGTTAACTGCACAGAAGTCCCTGTTGCTATTCATGCAGATCAACTTACTCCTACTTGGCGTGTT
rCOV2-ΔORF3a	GACTCTGAGCCAGTGCTCAAAGGAGTCAAATTACATTACACATAAGCACAAGCTGATGAGTACGAACTTATGTACTCATTCGTTTCGGAAGAGACAGGTACGTTAATAGT TAATAGCGTACTTCTTTTT
rCOV2-ORF8-FLAG	ATGAAATTTCTTGTTTTCTTAGGAATCATCACAACTGTAGCTGCATTTCACCAAGAATGTAGTTTACAGTCATGTACTCAACATCAACCATATGTAGTTGATGACCCGTGTC CTATTCACTTCTATTCTAAATGGTATATTAGAGTAGGAGCTAGAAAATCAGCACCTTTAATTGAATTGTGCGTGGATGAGGCTGGTTCTAAATCACCCATTCAGTACATCG ATATCGGTAATTATACAGTTTCCTGTTTACCTTTTACAATTAATTGCCAGGAACCTAAATTGGGTAGTCTTGTAGTGCGTTGTTCGTTCTATGAAGACTTTTTAGAGTATCAT GACGTTCGTGTTGTTTTAGATTTCATCGGCGGAGGTGGTTCTGACTACAAAGACCATGACGGTGATTATAAAGATCATGACATCGATTACAAGGATGACGATGACAAGTA AACGAACAAACTAAAATGTCT
rCoV2-link-Omicron BA.1(BA.5 S) S	GGTAACAAACCTTGTAATGGTGTTGCAGGTGTTAATTGTTACTTTCCTTTACAATCATATGGTTTCCGACCCACTTATGGTGTTGGTCAC
rCoV2-link-Omicron BA.1(BA.5 S) M	ATGGCAGGTTCCAACGGTACTATTACCGTTGAAGAGCTTAAAAAGCTCCTTGAAGAATGG
rCoV2-link-Omicron BA.5(BA.1 S) S	GGTAACAAACCTTGTAATGGTGTTGCAGGTTTTAATTGTTACTTTCCTTTACGATCATATAGTTTCCGACCCACTTATGGTGTTGGTCAC
rCoV2-link-Omicron BA.5(BA.1 S) M	ATGGCAAATTCCAACGGTAYTATTACCGTTGAAGAGCTTAAAAAGCTCCTTGAAGAATGG
rCoV2-link-Omicron BA.5(Wuhan S) S	GGTAGCACACCTTGTAATGGTGTTGAAGGTTTTAATTGTTACTTTCCTTTACATTCATATGGTTTCCAACCCACTAATGGTGTTGGTTAC
rCoV2-link-Omicron BA.5(Wuhan S) M	ATGGCAAATTCCAACGGTACTATTACCGTTGAAGAGCTTAAAAAGCTCCTTGAAGAATGG
BA.5 Δ3	ACGACTCTGAGCCAGTGCTCAAAGGAGTCAAATTACATTACACATAAGCACAAGCTGATGAGTACGAACTTATGTACTCATTCGTTTCGGAAGAGATAGGTACGTTAATA
BA.5 Δ3 M	ATGGCAAATTCCAACGGTACTATTACCGTTGAAGAGCTTAAAAAGCTCCTTGAAGAATGG
XBB Δ3	ACGACTCTGAGCCAGTGCTCAAAGGAGTCAAATTACATTACACATAAGCACAAGCTGATGAGTACGAACTTATGTACTCATTCGTTTCGGAAGAGATAGGTGCGTTAATA AAT
XBB Δ3 M	ATGGCAGATTCCAACGGTACTATTACCGTTGAAGAGCTTAAAAAGCTCCTTGAAGAATGG

Table S5. Primers used for the generation of recombinant SARS-CoV-2 virus.

Fragment	Primer	Sequence 5' to 3'	Amplicon Size
A1	F CMV	CGATGTACGGGCCAGATATACG	4694
	R A1-A2	GGCAGAATCTGGATGAAGATTGCC	
A2	F A2-A1	TGTGTTGAAGAAGTTACAACAACTCTG	4672
	R A-B	GTGTTATTAAATAGAAAATAGCAGCAACAAAAAGGAACACAA GTGTAACTTTAATTAACTGCTTCAACC	
B1	F B-A	GCACTTAAGGGTGGTAAAATTGTTAATAATTGGTTGAAGCA GTTAATTAAAGTTACACTTGTGTTCC	3292
	R B1-B2	CTACTTTGATACAAGGTTTGCCACC	
B2	F B2-B1	CACAGGGACTACTCCCACCC	3415
	R B-C	AAACTGTCTATTGGTCATAGTACTACAGATAGAGACACCAG CTACGGTGCGAGCTCTATTCTTTGCAC	
C1	F C-B	TATAACTCAAATGAATCTTAAGTATGCCATTAGTGCAAAGAA TAGAGCTCGCACCGTAGCTGGTG	3048
	R C1-C2	GTAGGATGTAACCCAGTGATTACC	
C2	F C2-C1	TTTACAAGTCTTGAAATTCCACGTAGG	3006
	R C-D	ATCACCAATCAAAGTTGAATCTGCATCAGAGACAAAGTCATT AAGATCTGAGTCGACAAGCAGCG	
D1	F D-C	TACAGCTGTTTTAAGACAGTGGTTGCCTACGGGTACGCTGC TTGTCGACTCAGATCTTAATGACTTTGTC	4622
	R D1-D2	CGAAAGGGAGTGAGGCTTGTATC	
D2	F D2-D1	GGAACTGTAACTTTGAAGCAAGGTG	4707
	R SV40	GCGGCCGCCAGACATGATAAG	
A	F CMV	CGATGTACGGGCCAGATATACG	9264
	R A-B	GTGTTATTAAATAGAAAATAGCAGCAACAAAAAGGAACACAA GTGTAACTTTAATTAACTGCTTCAACC	
B	F B-A	GCACTTAAGGGTGGTAAAATTGTTAATAATTGGTTGAAGCA GTTAATTAAAGTTACACTTGTGTTCC	6614
	R B-C	AAACTGTCTATTGGTCATAGTACTACAGATAGAGACACCAG CTACGGTGCGAGCTCTATTCTTTGCAC	
C	F C-B	TATAACTCAAATGAATCTTAAGTATGCCATTAGTGCAAAGAA TAGAGCTCGCACCGTAGCTGGTG	5947
	R C-D	ATCACCAATCAAAGTTGAATCTGCATCAGAGACAAAGTCATT AAGATCTGAGTCGACAAGCAGCG	
D	F D-C	TACAGCTGTTTTAAGACAGTGGTTGCCTACGGGTACGCTGC TTGTCGACTCAGATCTTAATGACTTTGTC	9229

	R SV40	GCGGCCGCCAGACATGATAAG	
D1 614	F D-C		2535
	R G614D	GAATAGCAACAGGGACTTCTGTGCAGTTAACATCCTGATAA AGAACAGCAACCTGGTTAGAAGTATTTGTTCC	
D2 614	F G614D	AACACCAGGAACAAATACTTCTAACCAGGTTGCTGTTCTTTA TCAGGATGTTAACTGCACAGAAGTCCCTGTTGCTATTC	6774
	R SV40		
D1 501	F D-C		2212
	R N501Y	AGTTCAAAAGAAAGTACTACTACTCTGTATGGTTGGTAACCA ACACCATAAGTGGGTTGGAAACCATATG	
D2 501	F N501Y	AAGGTTTTAATTGTTACTTTCCTTTACAATCATATGGTTTCCA ACCCACTTATGGTGTTGGTTACCAACCATAC	7117
	R SV40		
D1 Δorf3a	F D-C		4495
	R Δorf3a	CCTGTCTCTTCCGAAACGAATGAGTACATAAGTTCGTACTC ATCAGCTTGTGCTTATGTGTAATGTAATTTGACTCC	
D2 Δorf3a	F Δorf3a	ACGACTCTGAGCCAGTGCTCAAAGGAGTCAAATTACATTAC ACATAAGCACAAGCTGATGAGTACGAACTTATGTACTC	3912
	R SV40		
D1 orf8-3xFLAG	F D-C		7356
	R orf8-3xFLAG	TTACTTGTCATCGTCATCCTTGTAATCGATGTCATGATCTTT ATAATCACCGTCATGGTCTTTGTAGTCAGAACCACCTCCGC CGATGAAATCTAAAACAACACG	
D2 orf8-3xFLAG	F orf8-3xFLAG	GGCGGAGGTGGTTCTGACTACAAAGACCATGACGGTGATT ATAAAGATCATGACATCGATTACAAGGATGACGATGACAAG TAAACGAACAAACTAAAATGTCTG	1873
	R SV40		
linker	F linker	AGGCCACGCGGAGTACGATCGAGTGTACAGTGAACAATGC TAGGGAGAGCTGCC	1106
	R linker	CAGCCGAGTGACAGCCACAC	
A1-linker	F A-linker	GTTTAGTGAACCGTATATTAGGTTTATACCTTCCCAGGTAAC AAACC	4083
	R A1-A2		
D2-linker	F D2-D1		4451
	R D-linker	TTTGTCATTCTCCTAAGAAGCTATTAAAATCACATG	
D1 500	F D-C		500
	R 500	TCATGTCAAATAAAGAATAGGAAGACAACTG	
D2 500	F 500	CCACGCGAACAAATAGATGGTTATG	8829
	R SV40		

Table S6. PCR settings for individual fragments.

Fragment	Cycling Conditions	Elongation
A	5 cycles at 57 °C<br>20 cycles at 65 °C	4 min 40 s
B	5 cycles at 47 °C<br>20 cycles at 65 °C	3 min 20 s
C	5 cycles at 54 °C<br>20 cycles at 65 °C	3 min
D	5 cycles at 50 °C<br>20 cycles at 65 °C	4 min 20 s
A1	5 cycles at 52 °C<br>20 cycles at 64 °C	2 min 30 s
A2	5 cycles at 52 °C<br>20 cycles at 64 °C	2 min 30 s
B1	5 cycles at 48 °C<br>20 cycles at 63 °C	1 min 45 s
B2	5 cycles at 48 °C<br>20 cycles at 63 °C	1 min 45 s
C1	5 cycles at 48 °C<br>20 cycles at 63 °C	1 min 45 s
C2	25 cycles at 61 °C	1 min 45 s
D1	25 cycles at 63 °C	2 min 30 s
D2	5 cycles at 52 °C<br>20 cycles at 64 °C	2 min 30 s
linker	25 cycles at 58 °C	35 s
